# FIT calculator: a multi-risk prediction framework for medical outcomes using cardiorespiratory fitness data

**DOI:** 10.1038/s41598-024-59401-z

**Published:** 2024-04-16

**Authors:** Radwa Elshawi, Sherif Sakr, Mouaz H. Al-Mallah, Steven J. Keteyian, Clinton A. Brawner, Jonathan K. Ehrman

**Affiliations:** 1 Institute of Computer Science, University of Tartu, Tartu, Estonia; 2 Houston Methodist DeBakey Heart & Vascular Center, Houston, TX USA; 3 Division of Cardiovascular Medicine, Henry Ford Hospital, 6525 Second Ave., Detroit, MI 48202 USA

**Keywords:** Prediction model, Classification techniques, Interpretability, Automatic algorithm selection, Hyperparameter optimization, Health care, Computer science

## Abstract

Accurately predicting patients' risk for specific medical outcomes is paramount for effective healthcare management and personalized medicine. While a substantial body of literature addresses the prediction of diverse medical conditions, existing models predominantly focus on singular outcomes, limiting their scope to one disease at a time. However, clinical reality often entails patients concurrently facing multiple health risks across various medical domains. In response to this gap, our study proposes a novel multi-risk framework adept at simultaneous risk prediction for multiple clinical outcomes, including diabetes, mortality, and hypertension. Leveraging a concise set of features extracted from patients' cardiorespiratory fitness data, our framework minimizes computational complexity while maximizing predictive accuracy. Moreover, we integrate a state-of-the-art instance-based interpretability technique into our framework, providing users with comprehensive explanations for each prediction. These explanations afford medical practitioners invaluable insights into the primary health factors influencing individual predictions, fostering greater trust and utility in the underlying prediction models. Our approach thus stands to significantly enhance healthcare decision-making processes, facilitating more targeted interventions and improving patient outcomes in clinical practice. Our prediction framework utilizes an automated machine learning framework, Auto-Weka, to optimize machine learning models and hyper-parameter configurations for the simultaneous prediction of three medical outcomes: diabetes, mortality, and hypertension. Additionally, we employ a local interpretability technique to elucidate predictions generated by our framework. These explanations manifest visually, highlighting key attributes contributing to each instance's prediction for enhanced interpretability. Using automated machine learning techniques, the models simultaneously predict hypertension, mortality, and diabetes risks, utilizing only nine patient features. They achieved an average AUC of 0.90 ± 0.001 on the hypertension dataset, 0.90 ± 0.002 on the mortality dataset, and 0.89 ± 0.001 on the diabetes dataset through tenfold cross-validation. Additionally, the models demonstrated strong performance with an average AUC of 0.89 ± 0.001 on the hypertension dataset, 0.90 ± 0.001 on the mortality dataset, and 0.89 ± 0.001 on the diabetes dataset using bootstrap evaluation with 1000 resamples.

## Introduction

Predictive machine learning modeling serves as a powerful tool for extracting actionable insights from extensive clinical datasets, catering to various healthcare applications. Within the medical literature, significant attention has been devoted to disease risk prediction. Typically, the development of disease risk prediction models leverages cutting-edge machine learning algorithms, utilizing many features extracted from Electronic Health Records (EHRs). These models are primarily designed to predict individual diseases^1^. However, in real-world clinical scenarios, patients often present with multiple co-existing conditions, such as hypertension, diabetes, heart failure, obesity, and depression. This complexity necessitates a shift from single-risk prediction models to more comprehensive approaches that consider the interplay of multiple clinical outcomes. Instead of focusing solely on predicting a single disease, these multi-outcome prediction models aim to identify the most relevant features across various health conditions and train machine learning models accordingly. By adopting a holistic perspective on patient health, these multi-outcome prediction models offer a more nuanced understanding of disease dynamics and enable healthcare providers to deliver personalized and tailored interventions. Thus, there is a growing need to develop and deploy predictive modeling techniques that account for the multifaceted nature of clinical outcomes, ultimately enhancing the delivery of patient-centered care.

Overall, two primary obstacles hinder the widespread adoption of machine learning within the healthcare sector. The first hurdle lies in selecting the optimal machine learning algorithm and tuning its associated hyperparameters. This process is intricate, iterative, and time-consuming. The second challenge arises from medical staff's difficulty in comprehending and consequently trusting the predictions generated by machine learning models. This lack of understanding stems from a limited grasp of the primary features or health factors influencing the model's output predictions. As a result, there has been a significant focus on explaining the predictions of machine learning models in recent years. Numerous techniques have emerged to address this need, offering explanations for both global and local interpretations of complex machine learning models^2,3^. These efforts aim to bridge the gap in understanding between medical staff and machine learning models, ultimately enhancing trust and facilitating the integration of these models into clinical practice.

The machine learning modeling process is inherently iterative, as there is no single model that universally achieves the highest performance across all tasks. Consequently, it is common practice in machine learning to experiment with a multitude of models, each with different hyperparameter configurations^4^. This exploration is critical, as the choice of model and hyperparameter settings can profoundly impact model performance. For instance, research conducted using Weka, a popular machine learning framework, has demonstrated significant variability in accuracy across different machine learning algorithms^5^. On average, accuracy varied by 46% across 39 algorithms on 21 datasets and by 94% on a single dataset^5^. Moreover, for commonly used algorithms such as support vector machine and random forest, the average accuracy change exceeded 20% across 14 out of the 21 datasets^5^. This underscores the importance of careful model selection and hyperparameter tuning in achieving optimal performance. As a response to this challenge, considerable attention has been devoted to automating the process of machine learning algorithm selection and hyperparameter tuning. Several tools have emerged to address this need, including Auto-Weka and SmartML^6,7^. These automated tools have demonstrated their efficacy in achieving superior performance compared to models tuned manually by human experts^6^.

### Aims

In this study, we developed a multi-risk prediction framework for concurrently predicting the risk of mortality^8^, hypertension^9^, and diabetes^10^ using a minimum number of features extracted from the patients’ Cardiorespiratory Fitness Data^11^. In particular, the Auto-Weka framework has been used for choosing the best-performing model with the best set of hyper-parameters for the different medical outcomes. In addition, we explain each of the predicted outcomes in the form of the main health factors that contribute to each prediction using the local machine learning technique, SHAP TreeExplainer^12^.

## Material and methods

### Study setting and population

The FIT Project encompasses a cohort of 69,885 patients who underwent physician-referred treadmill stress testing at Henry Ford Health System-affiliated hospitals and ambulatory care centers in metropolitan Detroit, Michigan, spanning the years 1991–2009^11^. These medical facilities are integral components of a large, vertically integrated organization that delivers healthcare services and offers a managed care insurance plan. Data pertaining to treadmill stress testing, medical history, and medication were collected by exercise physiologists and nurses during the testing process and subsequently entered into a shared clinical reporting tool. This tool serves the dual purpose of generating clinical reports and directly populating the system's Electronic Medical Record (EMR). Supplemental clinical information and longitudinal follow-up data regarding cardiovascular outcomes were sourced from the EMR and administrative databases, which are uniformly shared across the system's affiliated subsidiaries. The FIT Project received approval from the Henry Ford Health System institutional review board, and all methodologies were executed in strict adherence to relevant guidelines and regulations.

### Machine learning pipeline

**Figure 1:**
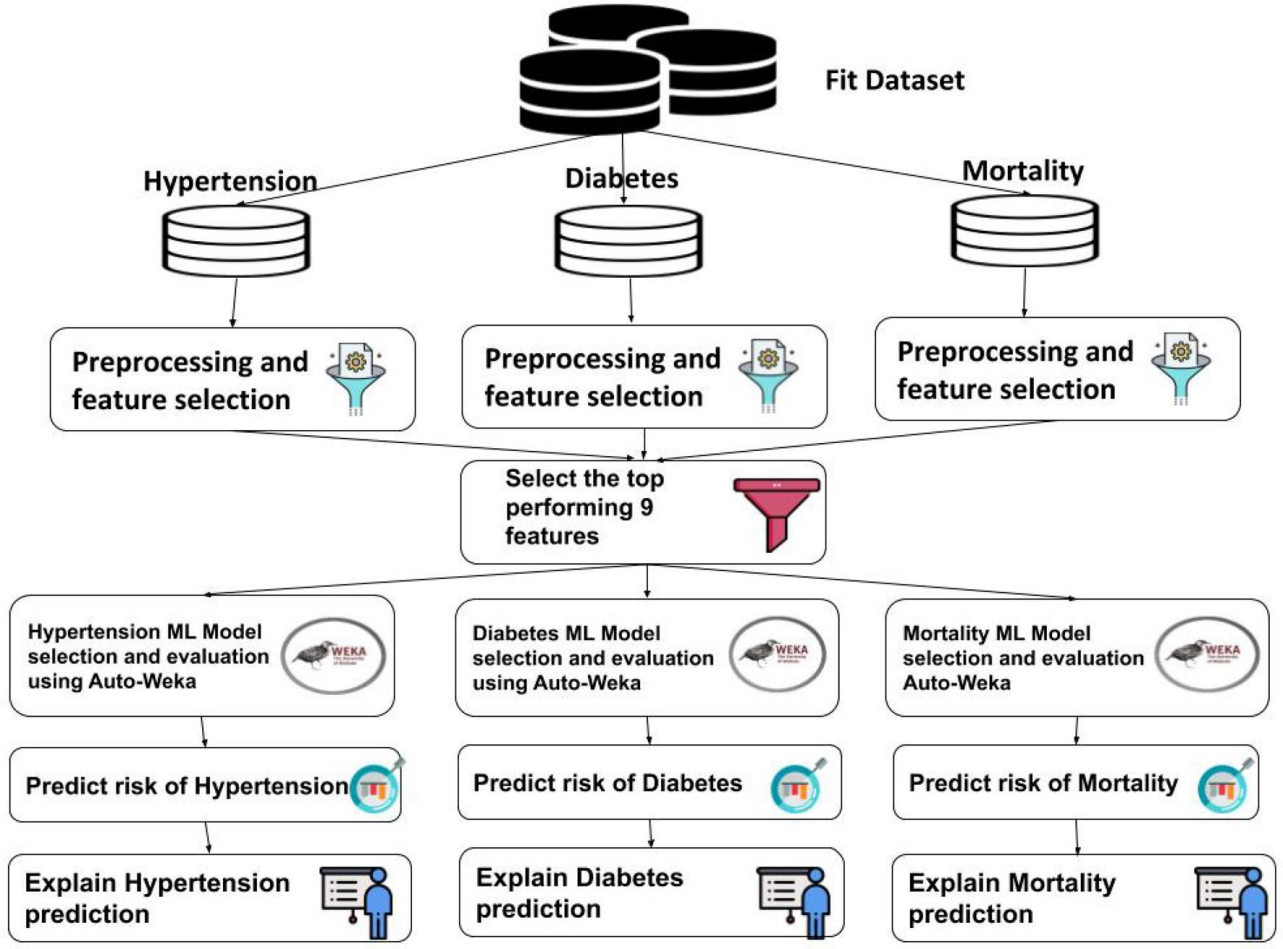
Machine learning pipeline for predicting and explaining the risk of mortality, hypertension and diabetes.

In the following, we explain the main components of the machine learning pipeline used to predict and explain the risk of mortality, hypertension, and diabetes as shown in Fig. 1.

#### Data extraction and inclusion

The dataset of our predictive models has been extracted from the Henry Ford ExercIse Testing (FIT) Project and the cohorts used in predicting the risk of mortality, hypertension, and diabetes are subsets of the main cohort. The cohort used in predicting mortality includes 34,212 patients who completed a 10-year follow-up. Note that we excluded from the original registry of the FIT project the patients with known coronary artery disease or heart failure at the time of the exercise test or those with less than 10-year follow-up. After a follow-up duration of 10 years, a total of 3921 patients (11.5%) died as verified by the national social security death index. All included patients have a social security number and were accounted for. In this study, we have classified the patients into two categories: low risk of all-cause mortality (ACM) and high risk of ACM. In particular, patients were considered to have a high risk for ACM if the predicted event rate is more than or equal to 3%. The cohort used in predicting the risk of diabetes is a subgroup of the entire cohort that included 32,555 patients who completed a 5-year follow-up for diabetes incidence. The dataset contains demographic characteristics, disease history, medication use history, and exercise test data for 62 attributes. The cohort used in predicting the risk of hypertension included 23,095 patients who completed a 10-year follow-up. The data set includes 43 attributes containing information on vital signs, diagnosis, and clinical laboratory measurements. The data set contains 23, 095 individuals (12,694 males (55%) and 10,401 (45%) females) with ages that range between 17 and 96. Half of the patients have a family history of cardiovascular diseases. During the 10-year follow-up, around 35% of the patients experienced hypertension. Male hypertension patients represent around 55% of the total hypertension patients while female patients represent around 44% of the total hypertension patients.

#### Data preprocessing

Data preprocessing constitutes a crucial component of the machine learning pipeline, exerting a substantial influence on the performance of the resulting models. Data preprocessing encompasses several key tasks, including handling missing values, removing outliers, sampling, and discretization. For each dataset utilized in our study, we applied consistent preprocessing methodologies, mirroring those employed in our prior research focused on predicting the risk of mortality^8^, hypertension^9^, and diabetes^10^. This standardized approach ensures comparability across datasets and maintains the integrity of our analytical framework.

#### Feature selection

Feature selection plays a critical role in the machine learning pipeline for several reasons. Firstly, it helps to mitigate the risk of overfitting by reducing the dimensionality of the data used to train the model. Additionally, it contributes to optimizing computational resources by reducing the time and space required for model building and training. To initiate the feature selection process, we began with the set of features utilized in our previous research focused on predicting the risk of mortality, hypertension, and diabetes. These features were initially chosen based on their information gain, which was assessed using the InfoGain module of the Weka software^13^. For further details on the feature extraction process, we refer the interested reader to^8–10^. The selected features employed in predicting the risk of diabetes encompassed *age, heart rate, METS, resting systolic blood pressure, resting diastolic blood pressure, sedentary lifestyle, race, obesity, hypertension, % HR achieved, hyperlipidemia, aspirin use, and family history of premature coronary artery disease*^10^. For predicting the risk of mortality, features included *age, METS, % HR achieved, history of hypertension, reason for test, resting systolic blood pressure, history of diabetes, diuretic use, atrial fibrillation, hypertension medication use, diabetes medication use, family history of CHD, history of smoking, race, and sex*^8^. In predicting the risk of hypertension, features comprised *age, METS, resting systolic blood pressure, peak diastolic blood pressure, resting diastolic blood pressure, family history of premature coronary artery disease, reason for test, history of diabetes, percentage HR achieved, race, history of hyperlipidemia, aspirin use, and hypertension response*^9^. Subsequently, we aimed to identify the smallest set of features that yielded the highest performance across all three models, restricting the maximum number of features to nine. This was achieved by first selecting the common features across the three models and then exploring all possible combinations of these features. Each combination was employed to train a separate model for predicting each outcome (diabetes, hypertension, and mortality) using Auto-Weka. The combination of features that achieved the best-combined performance across the three models was ultimately selected. Our findings revealed that the optimal combination of features comprised *age, race, METS, resting systolic blood pressure, resting diastolic blood pressure, reason for test, history of hyperlipidemia, % heart rate achieved, and family history of coronary artery disease.*

#### Machine learning algorithm selection

T*o* develop high-quality performing machine learning models, data scientists traditionally invest significant effort in manually selecting and fine-tuning numerous machine learning algorithms. This process is inherently complex and time-consuming. Consequently, there is a growing emphasis on automating model-building processes to minimize human intervention. In our study, we leveraged Auto-Weka to train and evaluate models aimed at predicting the risk of diabetes, hypertension, and mortality. Auto-Weka is specifically designed to address the challenges of model selection and hyperparameter tuning by treating the entire WEKA framework as a unified, highly parametric machine learning framework. It employs Bayesian optimization techniques to identify robust model configurations for new datasets. Our approach involved running Auto-Weka for 24 h to identify the optimal model and hyperparameter settings for each of the three outcomes (diabetes, mortality, and hypertension). For each outcome, Auto-Weka evaluated approximately 2000 combinations of machine learning algorithms and hyperparameters. Subsequently, we report the top models selected by Auto-Weka based on their performance metrics, particularly the Area Under the Curve (AUC), for each medical outcome. Notably, Auto-Weka consistently selected Random Forest as the top-performing model across all three outcomes. *Random Forest (RF)* is a classification algorithm that operates by constructing a multitude of decision trees during both training and testing phases, where the output class is determined as the mode of the classes predicted by individual trees^14^. Essentially, the decision tree model learns simple decision rules derived from the dataset’s features^15^. When classifying a new record based on its feature vector, the input vector is evaluated against each tree in the forest. Each tree casts a vote for a specific class label, and the final class label is determined by majority voting across all trees in the forest. Let N denote the number of records in the dataset, and M represent the number of input features utilized during model training. The construction of each tree in the forest follows these steps: (1) Randomly sample N records with replacement to create a training dataset for the tree. (2) At each node, select a subset of m << M features at random, and determine the optimal split based on these m features. Notably, the value of m remains constant throughout the tree's growth. (3) Grow each tree to its maximum depth. Random Forest typically delivers notable performance enhancements compared to using a single decision tree classifier. Moreover, RF is characterized by its expeditious classification capabilities and its effectiveness in handling unbalanced large datasets.

### Model evaluation and validation

To assess the unique performance of our ML methodology, we employed two traditional techniques: k-fold cross-validation^16^ and bootstrap sampling, as outlined by Steyerberg et al.^17^. The Bootstrap resampling method is utilized to estimate the performance of a model on a hypothetical test set when an explicit one is unavailable. It aids in preventing overfitting and enhances the stability of ML algorithms. In this validation technique, a series of artificial new datasets is created through random sampling with replacement from the original dataset. Each ML model is subsequently trained on one of these sampled datasets and assessed on the original dataset. This process is reiterated 1000 times^18^. K-fold cross-validation involves partitioning the dataset into 10 equal-sized subsets, commonly referred to as folds. During each iteration, one fold is reserved for testing the model, while the remaining nine folds are utilized for training. This process is repeated until each fold has been used for testing exactly once, ensuring that every instance in the dataset is included in the testing phase once and in the training phase nine times. The results obtained from each iteration, including various performance metrics, are then averaged to yield the final evaluation outcome. The 10-fold cross-validation method offers several advantages over a single hold-out set evaluator. Notably, it exhibits lower variance by averaging results across 10 different partitions. Consequently, it is less susceptible to biases that may arise from a single partitioning of the data for training and testing purposes. As a result, it is considered a more robust validation technique compared to the hold-out method, which randomly splits the data into a single set of training and validation datasets^19^. In the context of prediction outcomes, the result of the prediction process typically falls into one of the following four categories:

- True Positive (TP): is an outcome where the model correctly predicts patients who are at high risk.
- False Positive (FP): is an outcome where the model incorrectly predicts patients who are at high risk as low risk.
- True Negative (TN): is an outcome where the model correctly predicts patients who are at low risk.
- False Negative (FN): is an outcome where the model incorrectly predicts patients who are at low risk as high risk.
- Accuracy: is a measure of the overall correctness of the model. It is calculated as the ratio of the correctly predicted instances and the total number of predicted instances.
  $${\text{Accuracy }} = \, \left( {{\text{TP}} + {\text{TN}}} \right)/\left( {{\text{TP}} + {\text{FP}} + {\text{TN}} + {\text{FN}}} \right)$$
- Precision: It is a measure of the accuracy given that a specific class has been predicted.
  $${\text{Precision }} = {\text{ TP}}/\left( {{\text{TP }} + {\text{ FP}}} \right)$$
- Sensitivity: It is a measure of the proportion of actual positive instances that are correctly predicted.
  $${\text{Sensitivity }} = {\text{ TP}}/\left( {{\text{TP}} + {\text{FN}}} \right)$$
- *F-score*: It represents the harmonic mean of precision and sensitivity.
  $$F{ - }score = { 2 }*{\text{ TP }}/\left( {{ 2}*{\text{ TP}} + {\text{FP}} + {\text{FN}}} \right)$$
- *Root Mean Squared Error* (*RMSE*): It is defined as the square root of the mean square error that measures the difference between values predicted by the model and the actual values observed, where $y^{\prime}$ is a vector of *n* predictions and *y* is the vector of *n* observed (actual) values
  $$RMSE = \sqrt {\left( {\frac{1}{n}\mathop \sum \limits_{i = 1}^{n} \left( {y^{\prime}_{i} - y_{i} } \right)^{2} } \right)}$$
- *ROC*: Receiver Operating Characteristic (ROC) Curve is a way to quantify the diagnostic value of a test over its whole range of possible cutoffs for classifying patients as positive vs. negative. In each possible cutoff, the true positive rate and the false positive rate are calculated as the X and Y coordinates in the ROC Curve^13^.

All results of the different metrics are then averaged to return the final result.

### Explaining the outcome of the prediction model

Generally speaking, there exists a fundamental tradeoff between model performance and interpretability. While complex machine learning models often excel in terms of performance across various tasks, their intricate nature poses challenges for understanding and trust, particularly within critical domains such as medicine. To address this issue, machine learning interpretability has emerged as a crucial tool for enhancing trust in complex models deployed in sensitive domains. Machine learning interpretability can be defined as the extent to which a human can comprehend and make sense of the outcomes produced by a machine learning model. For instance, when a complex model identifies a patient as being at high risk of developing diabetes, medical practitioners are keen to understand the key features driving this prediction. This understanding is vital not only for fostering trust in the model's predictions but also for ensuring transparency and accountability in decision-making processes. Following the General Data Protection Regulation (GDPR), any predictions generated by automated systems must be accompanied by explanations^18^. This requirement underscores the importance of interpretability in machine learning models, particularly in domains where decisions have significant implications for individuals’ well-being and privacy.

In this study, we utilized TreeExplainer^12^, which facilitates the exact computation of Shapley values in low-order polynomial time by capitalizing on the internal structure of tree-based models. Shapley values typically necessitate summing terms over all possible feature subsets, but TreeExplainer streamlines this process by collapsing the summation into a series of calculations specific to each leaf in a tree. This approach represents a significant improvement in computational complexity compared to previous exact Shapley methods, achieving an exponential complexity reduction. To calculate the impact of a specific feature subset during the Shapley value computation, TreeExplainer leverages interventional expectations over a background dataset supplied by the user^20^. However, it can also circumvent the need for a user-supplied background dataset by relying solely on the path coverage information stored in the model, typically derived from the training dataset. This feature enhances the usability and applicability of TreeExplainer, making it a versatile tool for interpreting machine learning models.

## Results

**Figure 2:**
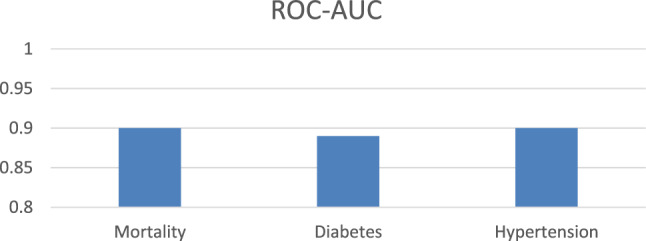
ROC-AUC performance of RF model for predicting the risk of mortality, diabetes and, hypertension through tenfold cross-validation.

The cohort of this study included 69,885 patients, of which 34,212 had all cause of mortality, 32,555 had diabetes and 23,095 had hypertension. Figure 2, shows the performance of the best performing algorithm selected by Auto-Weka for predicting the risk of hypertension, diabetes and mortality. The RF model selected by Auto-Weka achieves an average AUC of 0.90 ± 0.001 on the hypertension dataset, 0.90 ± 0.002 on the mortality dataset, and 0.89 ± 0.001 on the diabetes dataset based on 10-fold cross-validation. Using bootstrap evaluation with 1000 resamples, the RF model achieves an average AUC of 0.89 ± 0.001 on the hypertension dataset, 0.90 ± 0.001 on the mortality dataset, and 0.89 ± 0.001 on the diabetes dataset. These reported performances represent the average over 5 runs of the same experiment. The diabetes model was developed using only 7 attributes: *age, METS, resting systolic, resting diastolic, race, % heart rate achieved, hyperlipidemia,* and *family history of CAD.* For the hypertension model, 8 attributes were used: *age, resting systolic blood pressure, resting diastolic blood pressure, percent heart rate achieved, aspirin use, race, history of hyperlipidemia,* and *family history of CHD*. Similarly, the ML model for mortality prediction utilized 8 features: *age, resting systolic blood pressure, resting diastolic blood pressure, percent heart rate achieved, aspirin use, race, history of hyperlipidemia,* and *family history of CHD*. Table 1 summarizes the performance of the top-performing machine learning algorithm (RF) obtained from Auto-Weka for predicting the risk of diabetes, hypertension, and mortality using 10-fold cross-validation and bootstrap evaluation. The models evaluated using bootstrap sampling consistently demonstrated comparable performance to those evaluated through 10-fold cross-validation across all measured metrics, with minor variations observed in sensitivity, accuracy, precision, F-score, and RMSE. For instance, the random forest model for diabetes achieved similar performance with both cross-validation and bootstrap sampling, indicating consistent predictive ability. Similar patterns were observed for hypertension and mortality prediction models, underscoring their stability and reliability across evaluation methods.

**Table 1:** Performance of the top classifier (Random Forest) obtained from Auto-Weka using 10-fold cross-validation and bootstrap sampling for diabetes, hypertension, and mortality datasets.

	10-fold cross-validation			Bootstrap sampling		
	RF diabetes	RF hypertension	RF mortality	RF diabetes	RF hypertension	RF mortality
Sensitivity	0.82 ± 0.002	0.83 ± 0.003	0.92 ± 0.002	0.82 ± 0.002	0.84 ± 0.003	0.91 ± 0.002
Accuracy	0.82 ± 0.001	0.83 ± 0.002	0.92 ± 0.003	0.82 ± 0.001	0.83 ± 0.001	0.92 ± 0.003
Precision	0.82 ± 0.002	0.83 ± 0.001	0.92 ± 0.001	0.82 ± 0.001	0.82 ± 0.002	0.92 ± 0.001
F-Score	0.82 ± 0.002	0.82 ± 0.002	0.92 ± 0.002	0.83 ± 0.002	0.83 ± 0.003	0.93 ± 0.002
RMSE	0.35 ± 0.001	0.34 ± 0.002	0.25 ± 0.001	0.32 ± 0.002	0.33 ± 0.002	0.26 ± 0.002
AUC	0.89 ± 0.001	0.90 ± 0.001	0.90 ± 0.002	0.89 ± 0.001	0.89 ± 0.001	0.90 ± 0.001

**Figure 3:**
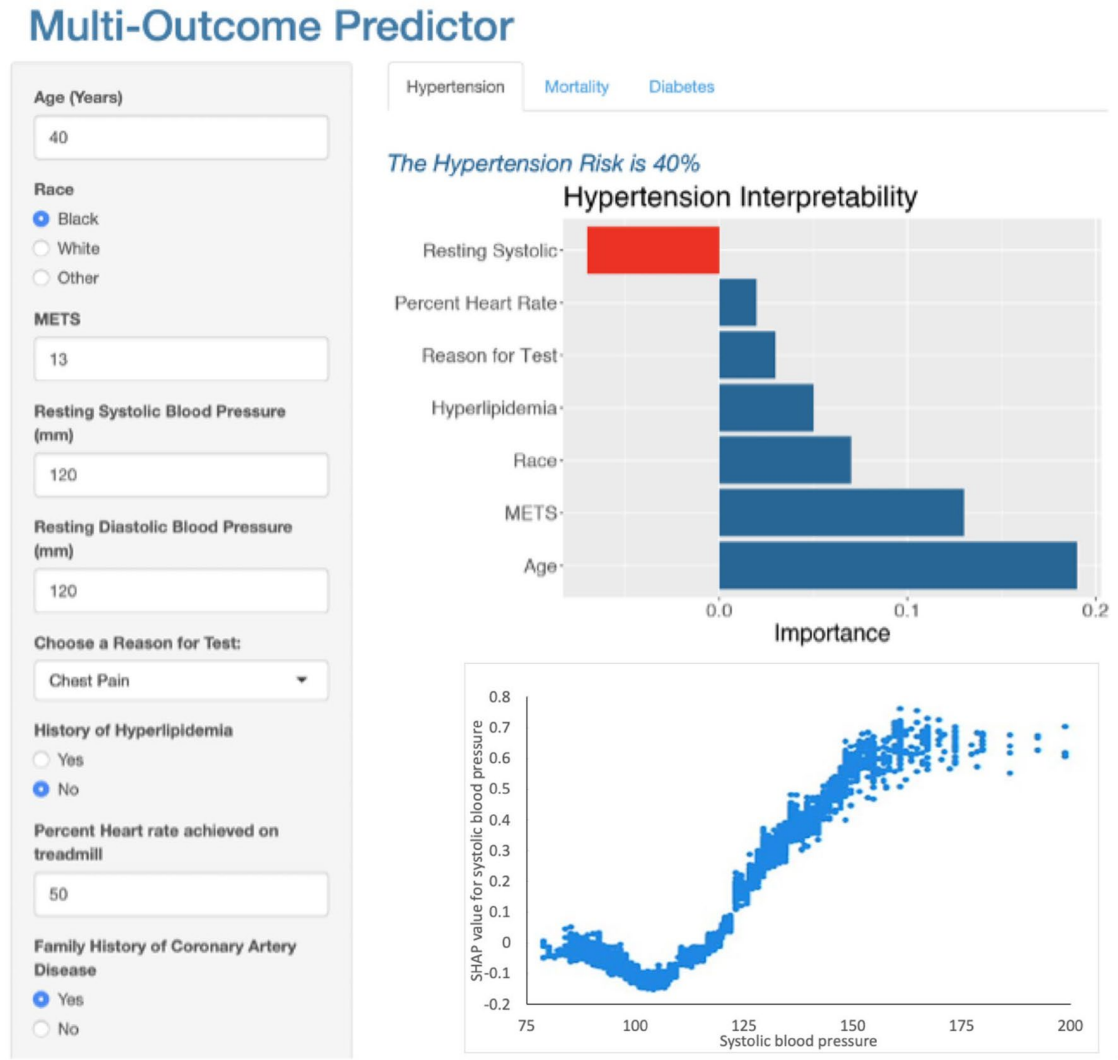
Screenshot of the multi-outcome predictor for a patient with the following features: Age = 40, Race = Black, METS = 13, Resting Systolic Blood Pressure = 120, Resting Diastolic Blood Pressure = 120, Reason for Test = Chest Pain, History of Hyperlipidemia = No, %Heart Rate Achieved = 50, Family History of Coronary Artery Disease = Yes. The patient's predicted risk of developing hypertension is 40%, accompanied by an explanation. The explanation chart indicates that resting systolic is the most contributed feature against the prediction while age and METS are the feature that contributes most toward the prediction.

**Figure 4:**
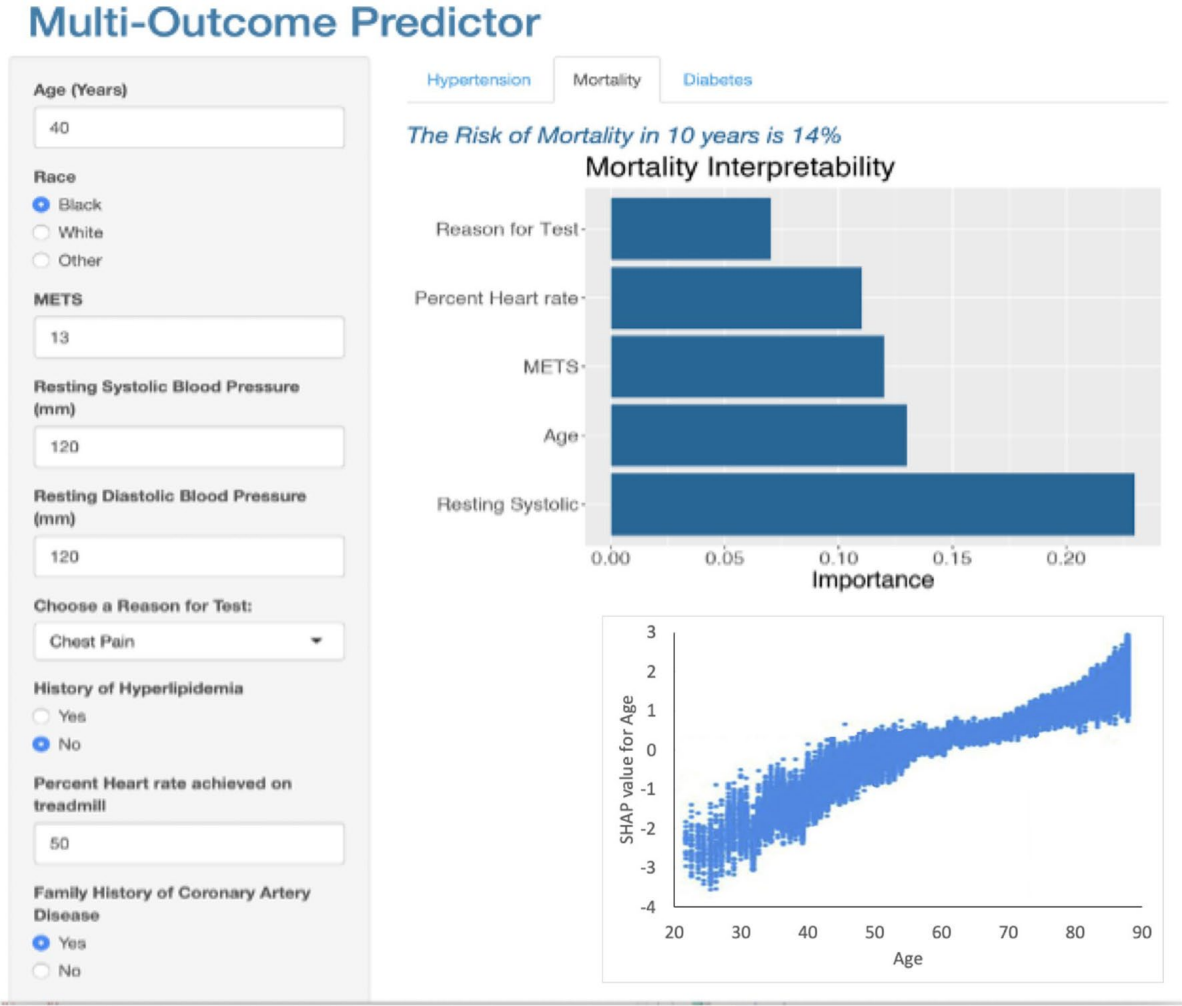
Screenshots of the multi-outcome predictor for a patient with the following features: Age = 40, Race = Black, METS = 13, Resting Systolic Blood Pressure = 120, Resting Diastolic Blood Pressure = 120, Reason for Test = Chest Pain, History of Hyperlipidemia = No, %Heart Rate Achieved = 50, Family History of Coronary Artery Disease = Yes. The patient's predicted risk of mortality is 14%, accompanied by an explanation. The explanation chart indicates that resting systolic blood pressure, age, and METS are the most influential factors contributing to this risk.

**Figure 5:**
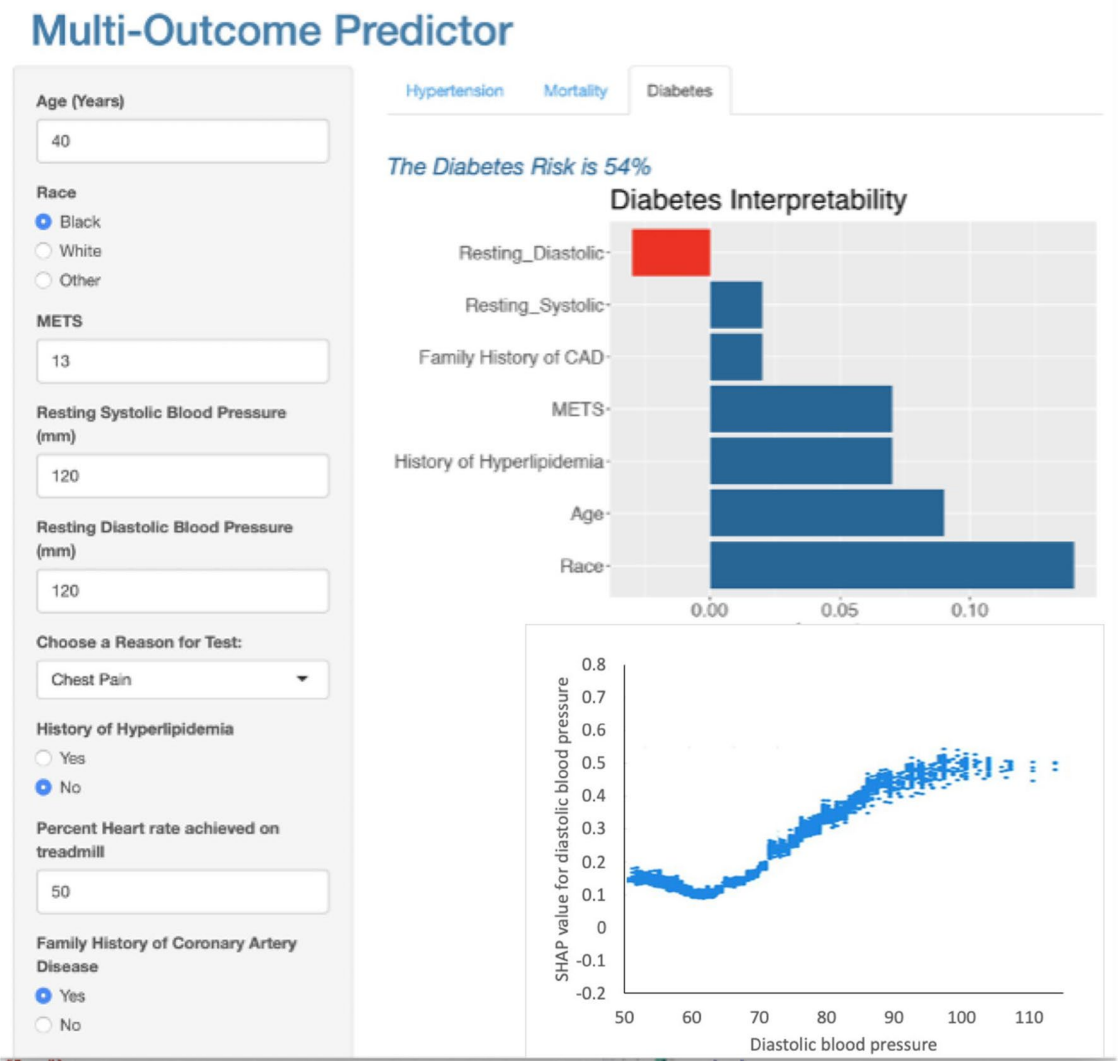
Screenshots of the multi-outcome predictor for a patient with the following features: Age = 40, Race = Black, METS = 13, Resting Systolic Blood Pressure = 120, Resting Diastolic Blood Pressure = 120, Reason for Test = Chest Pain, History of Hyperlipidemia = No, %Heart rate achieved = 50, Family History of Coronary Artery Disease = Yes. The patient has been predicted to have a 54% risk of diabetes, with an explanation for the predicted risk provided. It is noticeable from the explanation chart that race, age, and history of hyperlipidemia are the most contributing features toward the prediction while resting diastolic is the most contributing feature against the prediction.

We utilized the R Shiny package^21^ to develop a multi-outcome prediction application aimed at assisting physicians in timely risk assessment for mortality, hypertension, and diabetes, leveraging our prediction models with a limited number of features. Figures 3, 4, and 5 present screenshots of the FIT calculator, illustrating the prediction and explanation of risk for hypertension, mortality, and diabetes, respectively, based on specific patient features: age = 40, race = Black, METS = 13, resting systolic blood pressure = 120, resting diastolic blood pressure = 120, reason for test = Chest Pain, history of hyperlipidemia = No, %Heart rate achieved = 50, family history of Coronary Artery Disease = Yes. As depicted in Fig. 3, the patient has been predicted to have a 40% risk of developing hypertension, accompanied by an explanation of the predicted risk. The explanation chart highlights resting systolic blood pressure as the most influential feature in the prediction, while age and METS are identified as the primary contributing factors. By selecting a specific feature within the local explanation chart, users gain access to the dependence plot. Illustrated in Fig. 3, this plot, known as a SHAP dependence plot, effectively shows the influence of variables such as systolic blood pressure on hypertension prediction for individual patient cases in the dataset. Systolic blood pressure is plotted on the x-axis, while its impact on hypertension prediction is shown on the y-axis for each patient case, highlighting a significant increase in hypertension risk between systolic blood pressure values of 120 and 160 mmHg. In Fig. 4, the same patient has been predicted to have a 14% risk of mortality. Resting systolic blood pressure, age, and METS are identified as the most significant contributors to this risk. The SHAP dependence plot in Fig. 4 demonstrates how variables like age influence mortality prediction for individual patient cases, with age represented on the x-axis and its impact on mortality prediction on the y-axis, revealing a notable shift in mortality directionality from decline to increase between the ages of 48 and 77 years. Figure 5 indicates that the patient has been predicted to have a 54% risk of diabetes. Notably, race, age, and history of hyperlipidemia are identified as the most significant features contributing to this prediction, while resting diastolic blood pressure contributes against the prediction. The SHAP dependence plot in Fig. 5 illustrates the influence of variables such as diastolic blood pressure on diabetes prediction for individual patient cases, with diastolic blood pressure plotted on the x-axis. While higher diastolic blood pressure levels may correlate with increased diabetes risk, this relationship is influenced by various factors including age, BMI, family history, lifestyle, and other health markers, which should be considered comprehensively when assessing diabetes risk.

To assess the quality of explanations provided by the FIT calculator, we randomly selected 50 patient cases and presented the explanations generated by our tool to two physicians. The physicians were asked to evaluate whether the explanations aligned with their clinical intuition. Remarkably, our evaluation revealed that the explanations provided by the tool matched the physicians' intuition in 98% of the cases. This high level of agreement underscores the effectiveness and reliability of our interpretability approach in providing meaningful insights into model predictions.

## Discussion

Machine learning continues to revolutionize healthcare by facilitating the prediction of critical risk factors such as diabetes, hypertension, and mortality. Our study advances this field by developing machine learning models capable of predicting these risk factors using a minimal set of features, thereby enhancing model interpretability and practical utility in clinical settings. Leveraging the rich datasets from the FIT project, our approach not only aims for accurate predictions but also provides detailed explanations for each prediction instance, enhancing trust among healthcare professionals.

Comparing our contributions with existing literature reveals several distinctive aspects of our approach. While prior studies have explored machine learning-based risk prediction in healthcare, our emphasis on simplicity, interpretability, and prediction of multiple risk factors sets our study apart. For example, Brisimi et al.^22^ demonstrated the importance of model interpretability in healthcare applications by predicting chronic disease hospitalizations from electronic health records using an interpretable classification approach. In contrast, our study extends this approach to predict multiple risk factors while maintaining simplicity and interpretability.

Numerous studies have investigated the prediction of mortality risk using various methodologies. For instance, Knuiman et al.^23^ conducted a comparative analysis of four prediction techniques, with Cox regression emerging as the top performer. Similarly, Hsieh et al.^24^ evaluated machine learning models for predicting mortality risk in ICU settings, with the random forest model demonstrating superior performance. For diabetes risk prediction, decision tree models have shown promising results. For instance, a study comparing different machine learning techniques found that decision trees outperformed others in terms of accuracy^25^. Additionally, Cahn et al.^26^ demonstrated the prediction of diabetes risk using logistic regression and achieved commendable specificity rates. Various machine learning methods have also been explored for hypertension risk prediction. Ture et al.^27^ compared machine learning and statistical techniques, with artificial neural networks outperforming statistical methods. Another study developed a support vector machine-based risk calculator for hypertension prediction, achieving high discriminative performance^28^.

In our study, we observed consistent performance across different risk factors, with the Random Forest (RF) model achieving competitive results in predicting mortality, hypertension, and diabetes risks. The high AUC scores obtained suggest the strong discriminatory power of our models in distinguishing between positive and negative cases. However, it's important to critically interpret these results in the context of real-world applications. While high AUC scores indicate good model performance in controlled settings, translating these findings into meaningful improvements in clinical practice requires careful consideration of various factors such as model generalization, scalability, and impact on patient outcomes. The observed performance of our models can be attributed to several factors. Firstly, the careful selection of features based on domain knowledge and data-driven approaches ensures that the models capture relevant information for risk prediction. Additionally, the use of ensemble methods such as Random Forests enhances model robustness by aggregating predictions from multiple decision trees, thereby reducing the risk of overfitting. Moreover, the availability of rich and diverse datasets from the FIT project allows our models to learn complex patterns and relationships, contributing to their predictive performance.

## Data Availability

Due to ethical restrictions imposed by the Institutional Review Board of Henry Ford Health Systems, the data underlying this study are available upon request to interested researchers after receiving the Institutional Review Board’s approval. Informed consent was obtained from all human participants involved in the study. To apply for access to these data, interested researchers may query Dr. Tim Roehrs (troehrs1@hfhs.org) of Henry Ford Health Systems’ Institutional Review Board, or the Research Administration team (Research_Admin@hfhs.org).
